# Altered Plasma Acylcarnitines and Amino Acids Profile in Spinocerebellar Ataxia Type 7

**DOI:** 10.3390/biom10030390

**Published:** 2020-03-03

**Authors:** Rafael Nambo-Venegas, Claudia Valdez-Vargas, Bulmaro Cisneros, Berenice Palacios-González, Marcela Vela-Amieva, Isabel Ibarra-González, César M. Cerecedo-Zapata, Emilio Martínez-Cruz, Hernán Cortés, Juan P. Reyes-Grajeda, Jonathan J. Magaña

**Affiliations:** 1 Laboratory of Chronic Diseases Biochemistry, National Genomics Medicine Institute (INMEGEN), Mexico City 14610, Mexico; rafaelnambo@yahoo.com.mx; 2 Laboratory of Genomic Medicine, Department of Genetics, National Rehabilitation Institute (INR-LGII), Mexico City 14389, Mexico; cvaldezqbp@outlook.com (C.V.-V.); hcortes@inr.gob.mx (H.C.); 3 Department of Genetics and Molecular Biology, Center of Research and Advanced Studies (CINVESTAV-IPN), Mexico City 07360, Mexico; bcisnero@cinvestav.mx; 4 Scientific Bonding Unit, Medicine Faculty UNAM-INMEGEN, Mexico City 14610, Mexico; bpalacios@inmegen.gob.mx; 5 Laboratory of Inborn errors of metabolism, National Pediatrics Institute (INP), Mexico City 04530, Mexico; dravelaamieva@yahoo.com; 6 Institute of Biomedical Research, IIB-UNAM, Mexico City 04510, Mexico; icig@servidor.unam.mx; 7 Rehabilitation and Special Education Center of Veracruz (CRISVER-DIF), Xalapa 91097, Veracruz, Mexico; misael_207@hotmail.com (C.M.C.-Z.);

**Keywords:** spinocerebellar ataxia 7, CAG repeats, polyglutamine disease, metabolomics, biomarkers, mitochondrial disease, metabolic pathways

## Abstract

Spinocerebellar ataxia type 7 (SCA7), a neurodegenerative disease characterized by cerebellar ataxia and retinal degeneration, is caused by an abnormal CAG repeat expansion in the *ATXN7* gene coding region. The onset and severity of SCA7 are highly variable between patients, thus identification of sensitive biomarkers that accurately diagnose the disease and monitoring its progression are needed. With the aim of identified SCA7-specific metabolites with clinical relevance, we report for the first time, to the best of our knowledge, a metabolomics profiling of circulating acylcarnitines and amino acids in SCA7 patients. We identified 21 metabolites with altered levels in SCA7 patients and determined two different sets of metabolites with diagnostic power. The first signature of metabolites (Valine, Leucine, and Tyrosine) has the ability to discriminate between SCA7 patients and healthy controls, while the second one (Methionine, 3-hydroxytetradecanoyl-carnitine, and 3-hydroxyoctadecanoyl-carnitine) possess the capability to differentiate between early-onset and adult-onset patients, as shown by the multivariate model and ROC analyses. Furthermore, enrichment analyses of metabolic pathways suggest alterations in mitochondrial function, energy metabolism, and fatty acid beta-oxidation in SCA7 patients. In summary, circulating SCA7-specific metabolites identified in this study could serve as effective predictors of SCA7 progression in the clinics, as they are sampled in accessible biofluid and assessed by a relatively simple biochemical assay.

## 1. Introduction

Spinocerebellar ataxia type 7 (SCA7) is an inherited neurodegenerative disorder characterized by progressive degeneration of the cerebellum, gait ataxia, cone-rod retinal dystrophy, and progressive loss of central vision, which ultimately leads to complete blindness [1,2,3]. SCA7 is caused by a Cytosine-Adenine-Guanine (CAG) repeat expansion in the *ATXN7* gene coding region, which in turn leads to a polyglutamine (polyQ) expansion at the amino terminus of the encoded protein, ataxin-7 [4,5]. The polyQ tract ranges from 4 to 18 glutamines in healthy individuals and from 36 to 460 in affected subjects [6,7]. A correlation has been observed between the number of CAG repeats and the age at onset, with repeats below 46 CAGs corresponding to adult-onset (AO) and CAGs expansions above 70 repeats related to early-onset (EO) [3,8]. EO has a rapid and aggressive course of the disease, and it is commonly associated with regression of motor milestones. At the cellular level, different processes are altered in SCA7, including transcriptional regulation, oxidative stress, mitochondrial function, protein turnover, autophagy, and cell death [9,10,11,12,13,14,15,16,17,18].

The clinical spectrum of SCA7 is broader than previously recognized, with heterogeneous phenotypes originating from particular compositions of cerebellar and non-cerebellar symptoms. Apart from the CAG repeat length, environmental and genetic modifiers might contribute to SCA7 pathophysiology [8]; thus, identification of sensitive biomarkers will facilitate the monitoring of disease progression and assessing of treatments, which ultimately will improve clinical management of patients. Metabolomics has emerged as a powerful technique to identify potential biomarkers in neurodegenerative diseases [19,20,21] as alterations in brain function can be reflected in the metabolite composition of biofluids such as the serum, plasma, and cerebrospinal fluid (CSF) [22,23,24]. Indeed, recent reports have described the detection of some neurometabolites associated with specific changes in neuronal and astrocytic cells in patients with different SCAs [25].

In order to identify the metabolic pathways and potential biomarkers in SCA7, we carried out a metabolomics profiling of free carnitine, acylcarnitines (ACCs), and amino acids (AAs) in the patients’ plasma samples. We focused on the metabolites mentioned above because these are associated with mitochondrial dysfunction and oxidative stress [26,27,28], two main pathophysiological features of SCA7 [13,15,16]. We found diverse circulating metabolites to be altered in SCA7 patients and identified a signature of metabolites with the ability to discriminate between patients and healthy subjects. Moreover, we determined a second set of metabolites, whose altered levels appear to be associated with the specific disease phenotype, thereby serving potentially as biomarkers for disease severity progression.

## 2. Materials and Methods

### 2.1. Study Participants

Twenty patients with SCA7 and twenty age- and gender-matched healthy relative controls (confirmed by genetic testing) were recruited by the National Rehabilitation Institute (INR) and the Rehabilitation and Social Inclusion Center of Veracruz-DIF (CRISVER-DIF). Patients and healthy controls individuals had similar nourishment and lifestyle conditions. A complete medical history of each participant was obtained by structured interviews. Patients who had a clinical stroke, systemic disorders, or other brain diseases that could produce progressive neurodegeneration, as well as patients with secondary ataxias due to neoplasias, malformations, alcoholism, autoimmune or inflammatory diseases, neuropharmacological treatment, vascular pathology, and other non-genetic causes, were excluded. Likewise, participants with any of the following conditions were also excluded: fever, infection, pregnancy, dementia, alcohol abuse, liver damage, kidney failure, current therapy with immunosuppressive or anticonvulsant medicines or antioxidants, autoimmunological disorders, or other severe illnesses.

The study was approved by the INR Ethics/Research Committee, and informed consent from each participant was obtained.

### 2.2. Clinical Assessment

Patients were clinically examined according to the Mayo Clinic procedures [29]. Dysdiadochokinesia and dysmetria were identified through the examination of motor control of the limbs, while the detection of gait ataxia, tremor, or dysarthria was performed by the evaluation of cerebellar signs. Altered ocular movements were determined by the examination of the cranial nerve, while involuntary movements were evaluated by the exploration of extrapyramidal signs. Other clinical features analyzed included muscle strength and pathological reflexes. Information about the age at which the first symptom had appeared was provided either by the patients or their relatives. The severity of ataxia-associated features was evaluated with the Scale for the Assessment and Rating of Ataxia (SARA), while the assessment of extra-cerebellar symptoms was performed with the Inventory of Non-Ataxia Symptoms (INAS) [30,31]. Patients with SCA7 were classified into two clinical phenotypes: AO and EO. This categorization was based on the age at onset of the first referred symptoms (AO patients, > 20 years; EO patients, < 20 years) [3]. Commonly, EO patients exhibited visual symptoms (macular dysfunction or decreased visual acuity) within the first two decades of their life, while AO patients showed motor alterations as the first symptom until after the third decade of life.

### 2.3. Metabolomics Analysis

Fasting blood samples were collected from all subjects at the same time in the morning. Blood was collected by venipuncture and placed in EDTA tubes (BD Vacutainer, NJ, USA). Samples were centrifuged at 2,500 rpm for 10 min to obtain plasma, and the white buffy layer (leukocytes) was removed. Plasma samples were stored at −70 °C until analysis. A targeted metabolomics approach was employed to determine the concentration of AAs, free carnitine, and ACCs using electrospray tandem mass spectrometry. Plasma levels of metabolites were determined with a commercial kit (NeoBase Non-derivatized MS/MS Kit, Perkin Elmer, Waltham, MA, USA), following the fabricant´s specifications. In brief, 20 µL of plasma samples were dropped onto filter paper cards (Whatman 903™, Schleicher & Schüell, Dassel, Germany) and dried for 4 h at room temperature in a sterile environment. The resulting spot was precisely cut off in 2 mm circles and placed into a 96-well plate, and then 190 µL of extraction solution containing a mixture of 22 stable isotope-labeled internal standards were added. The plate was sealed, incubated under stirring (30 °C at 650 × g for 30 min), and then placed in a Waters autosampler. An HPLC pump (Waters 2795) was employed for the delivery of solvent, supplying a 0.1 mL/min stream of a mixture of acetonitrile:water (80:20 v:v%). Ten microliters of each sample were directly administered into the flow at 4-min intervals. A blank sample containing extraction solution and internal standards was included in each plate in triplicate, as reference. A Micromass Quattro instrument (Waters Inc., Milford, MA, USA) coupled to an ESI source in positive mode was employed. For desolvation and nebulization, nitrogen gas was utilized, while argon was employed as the collision gas.

### 2.4. Statistical Analysis

Means and standard deviations (SD) were calculated for the descriptive statistics of the demographic and clinical characteristics of subjects. The normality of variables was analyzed by the Kolmogorov-Smirnov test, while a Kruskal-Wallis test was performed to analyze differences in age and CAG repeats between AO and EO patients and healthy controls. Differences between AO and EO patients for age at onset of visual symptoms, age at onset of motor symptoms, age at onset of the first symptom, SARA, and INAS were calculated by the Mann-Whitney test or unpaired *t*-test. *P* values < 0.05 were considered significant in all cases. Partial Least Squares Discriminant Analysis (PLS-DA) was performed to identify independent predictors that best correlated with SCA7. In order to minimize the possibility that the observed separation on PLS-DA was by chance, permutation testing was carried out. This testing involved repeated (2000 times) data sampling, with different random labeling. A Variable Importance in Projection (VIP) plot was performed for ranking the metabolites based on their importance in discriminating study from healthy volunteers. VIP cutoff > 1.0 was selected since the number of variables in this study was less than 100. The differences between the group with SCA7 and healthy volunteers were evaluated through an analysis of Random Forest decision trees according to the distribution of the variables. In order to evaluate the association and contribution of the variables to differentiate the SCA7 group from the healthy volunteers, an analysis of Random Forest decision trees was performed according to different selection criteria: frequency and average. Based on the variables that presented a higher frequency or average, a model was generated that allowed classifying the group with SCA7. To evaluate the diagnostic power of each biomarker alone, we performed univariate Receiver Operating Characteristic (ROC) analysis on each biomarker in order to obtain its ROC curve, ROC Area Under the Curve (AUC), and standard error (SE) of the AUC and these were plotted in an R-environment. After univariate ROC analysis on each of the three markers, multivariate ROC analysis on each model was performed. Our goal was to select the panel with the highest ROC AUC. We performed a Metabolite Set Enrichment Analysis (MSEA) to confirm biologically meaningful patterns between healthy vs. SCA7 patients. Quantitative enrichment analysis (QEA) was performed using the “globaltest 3” package. All statistical analyses were performed in Prism version 6.01 (GraphPad Software, San Diego, CA, USA), R version 64, Metabo Analyst 4.0 (McGill University, Toronto, Canada), Stata version 13 (Stata Corporation, College Station, TX, USA), and statistical significance was assumed if the probability value was less than 0.05.

## 3. Results

### 3.1. Characteristics of Study Subjects

The study sample included 20 patients with SCA7 and 20 age- and gender-matched healthy relatives. Clinical characteristics and demographic features of the studied subjects are described in Table 1. Clinical features of patients included gait ataxia and cerebellar syndrome, hyperreflexia, dysmetria, dysdiadochokinesia, and visual impairment. No significant differences were observed in the demographic characteristics between the two groups (*p* > 0.05). Patients were categorized into EO and AO phenotypes (see Material and Methods); as expected, EO patients exhibited a more severe form of the disease and a larger CAG repeat tract than AO subjects.

### 3.2. Differential Metabolic Profile of ACCs and AAs in SCA7 Patients

**Figure 1:**
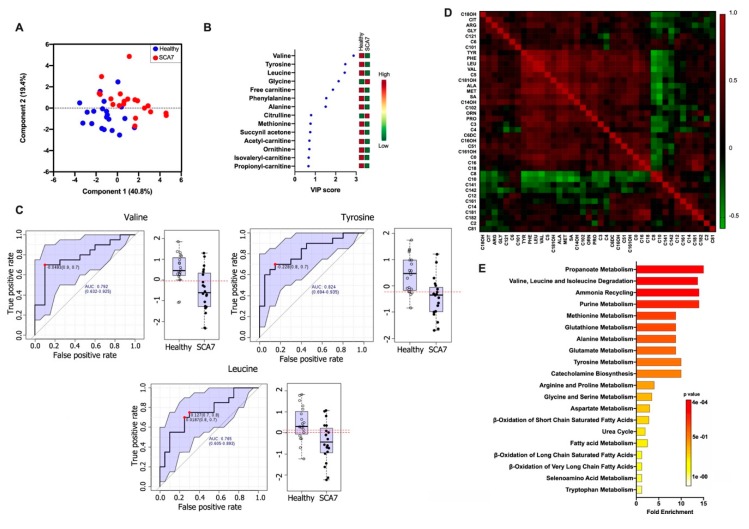
(**A**) Partial Least Squares Discriminant Analysis (PLS-DA) plot showing separation between groups; healthy group (blue circles) and SCA7 group (red circles). The explained variances are shown in brackets; (**B**) Variable Importance in Projection (VIP) analysis represents the relative contribution of metabolites to the variance between healthy controls and SCA7 patients. A high VIP score indicates a great contribution of the metabolites to the group separation. The green and red boxes on the right indicate whether metabolite concentration is increased (red) or decreased (green); (**C**) Receiver Operating Characteristics (ROC) curves of Valine, Tyrosine, and Leucine. The sensitivity is plotted on the y-axis, and the specificity is on the x-axis. The Area Under the Curve (AUC) is in blue. The right image is a boxplot of the two groups within the dataset. A horizontal red indicates the optimal cutoff; (**D**) Unsupervised clustering of Correlation heatmap; red and green colors indicate increased and decreased correlation, respectively. (**E**) Enrichment analysis using Metabolite Set Enrichment Analysis (MSEA) for metabolic pathways in SCA7.

To determine whether the profile of free carnitine, AACs, and AAs is altered in SCA7, plasma samples from 20 patients (10 EO and 10 AO) and 20 healthy relatives were analyzed using electrospray tandem mass spectrometry. A total of 49 metabolites were accurately identified and quantified, of which 21 metabolites were significantly different between patients and controls (*p* < 0.05). The predictive power of the metabolite profile to discriminate between patients and healthy controls was tested by the supervised PLS-DA approach. PLS-DA score plots significantly separated patients from healthy controls (Figure 1A) with an accuracy of 60.2% and with values of 0.80 and 0.61 for R2 and Q2, respectively. Permutation testing showed that separation between the groups was highly unlikely to be by chance (*p* < 0.0005), and the corresponding VIP plot showed that metabolites were responsible for the separation between patients and healthy controls. Interestingly, valine, tyrosine, leucine, glycine, free-carnitine, phenylalanine, and alanine showed VIP scores above 1.0 on VIP analysis, which made them potentially useful for discrimination between groups (Figure 1B). We next applied a Receiver Operating Characteristics (ROC) analysis, based on best predictor ratios, to assess the sensitivity and specificity of single metabolites. The ROC AUCs and standard errors were based on complete data from 40 samples; the AUC of each analyzed biomarker was more than 0.5, as expected. We obtained AUC values of 0.792, 0.824, and 0.765 for Valine, Tyrosine, and Leucine, respectively (Figure 1C).

Finally, unsupervised hierarchical clustering of correlation heatmap was obtained, which showed a decreased concentration of Tyrosine, Phenylalanine, Leucine, and Valine in SCA7 patients and that these amino acids correlated similarly with all metabolites studied (Figure 1D). Enrichment analysis using MSEA identified alterations in a variety of metabolic pathways in SCA7 (Figure 1E), including the metabolism of different amino acids, ammonia recycling, glutathione metabolism, catecholamine biosynthesis, β-oxidation of long-chain fatty acids and oxidation of branched-chain fatty acids, purine metabolism, and carnitine synthesis.

### 3.3. Assessment of ACCs and AAs as Disease Biomarkers for SCA7 by Multivariate Model and ROC Analyses

**Figure 2:**
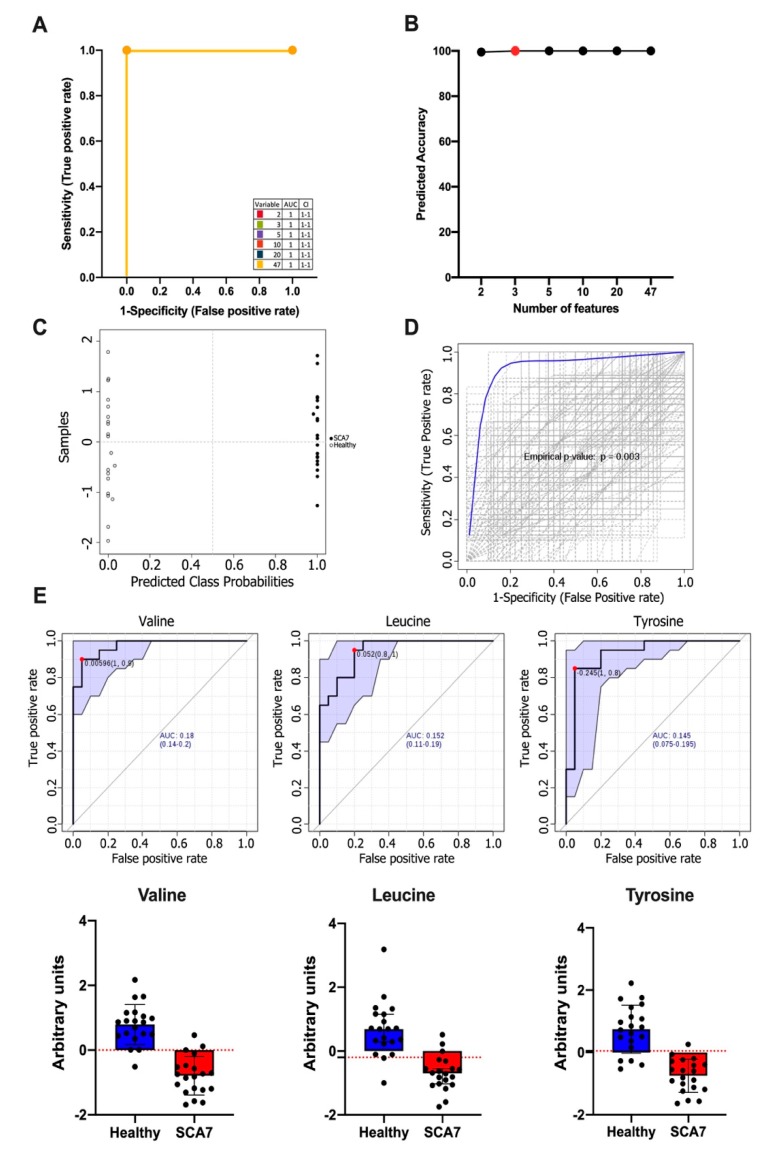
(**A**) ROC curves for all models are based on their average performance; (**B**) Predictive accuracy of biomarker models with an increasing number of features. The most accurate biomarker model is highlighted with a red dot; (**C**) Predicted class probabilities for all samples (healthy controls (open circle) and SCA7 patients (filled circle)) using the created biomarker model. Due to balanced subsampling, the classification boundary is at the center (x = 0.5, dotted line); (**D**) Permutations tests using the area under the ROC curve or the predictive accuracy of the model as a measure of performance. The plot shows the AUC of all permutations, highlighting the actual observed AUC in blue, along with showing the empirical *p*-value (*p* = 0.003); (**E**) ROC curves of Valine, Tyrosine, and Leucine. The sensitivity is plotted on the y-axis and the specificity on the x-axis. The Area Under the Curve (AUC) is in blue. The graphs under ROC curves correspond to a boxplot of the two experimental groups within the dataset. ROC analysis calculated by FRPmax (False positive rate).

We sought to identify specific metabolites that may serve as prognostic markers in SCA7, using a computational analysis based on Random Forest metabolite selection and regression analyses. Firstly, multivariate ROC analyses to correlate the concentration of metabolites with clinical features (age, gender, number of CAG repeats, age at onset of motor and visual symptoms, and SARA and INAS scores) were performed (Figure 2A). A model consisting of three metabolic variables (Valine, Leucine, and Tyrosine) was the best to discriminate between patients and healthy controls, with a prediction accuracy of 100% (Figure 2B). The combination of metabolite data with clinical characteristics is shown in Table S1. The predicted class probabilities using AUC scoring successfully classified cases (100%) and healthy controls (100%) (Figure 2C). Permutation test for the user-created biomarker model (Valine, Leucine, and Tyrosine) is plotted in Figure 2D, with an empirical *p*-value of 0.003. Based on both the random forest analysis and the regression approach, we obtained a robust diagnostic metabolite signature. Finally, ROC scores demonstrated that separately Valine (AUC = 0.820), Leucine (AUC = 0.848), and Tyrosine (AUC = 0.855), indeed, have the power to discriminate SCA7 patients from healthy controls (Figure 2E). It is evident that the presence of these three metabolites decreases considerably in the presence of SCA7 (Figure 2E) and confirms the diagnostic value of these metabolites for SCA7.

### 3.4. Differential Metabolic Profile between EO and AO Patients

**Figure 3:**
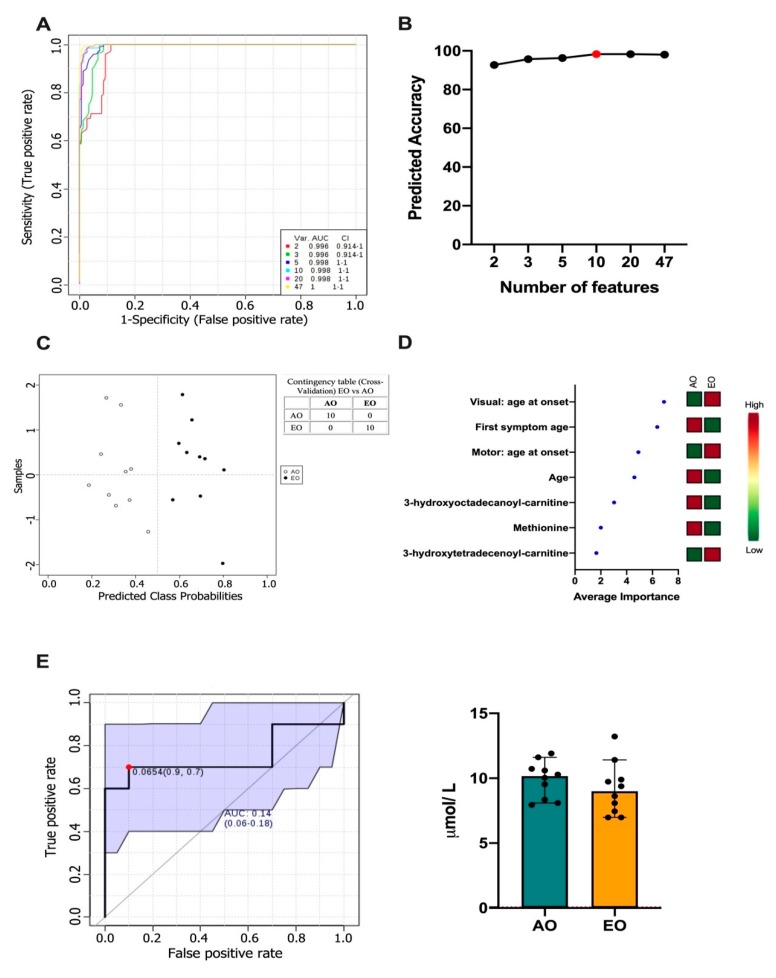
(**A**) ROC curves for all models based on its average performance; (**B**) Predictive accuracy of the biomarker models with an increasing number of features. The most accurate biomarker model is highlighted with a red dot; (**C**) Predicted class probabilities for all samples (Adult-Onset (open circle) and Early-Onset patients (filled circle)), using the created biomarker model. Because of a balanced subsampling, the classification boundary is at the center (x = 0.5, dotted line); (**D**) Plot of the most important features of a selected model, ranked from most to least important. (**E**) ROC curve of Methionine. The sensitivity is plotted on the y-axis and the specificity on the x-axis. The Area Under the Curve (AUC) is in blue. The image on the right corresponds to a boxplot of the two groups within the dataset. ROC analysis calculated by FRPmax (False positive rate).

Owing to the heterogeneous clinical presentation of SCA7, we next sought to identify specific metabolites that may serve as prognostic markers as well as differentiate between EO and AO phenotypes, using a computational analysis based on Random Forest metabolite selection and regression analyses. Firstly, multivariate ROC analyses based on the combination of clinical and metabolic variables were performed (Figure 3A and Table S2). A model that consisted of 10 variables was the best to discriminate between SCA7 phenotypes with a prediction accuracy of 98.3% (Figure 3B). The predicted class probabilities (average of the cross-validation) for each sample were calculated using the AUC-based best classifier (Figure 3C). The model successfully classified EO and AO phenotypes, as shown in the contingency table; ranking (from most to least important) of the characteristic that allowed discrimination between AO and EO patients is shown in Figure 3D.

Interestingly, apart from clinical variables (age of onset of visual and motor symptoms and patient age), the metabolites 3-hydroxyoctadecanoyl-carnitine (AC18OH), Methionine, and 3-hydroxytetradecanoyl-carnitine (AC14OH) could distinguish between disease phenotypes, thereby increasing the prognostic power of the model. Decreased levels of AC18OH and Methionine, as well as increased levels of AC14OH, were found in EO patients, compared to AO patients, as shown by VIP analysis. ROC curves confirmed the robust prognostic power of the metabolites’ signature, with AUC scores of 0.860, 0.920, and 0.990 for Methionine, AC14OH, and AC18OH, respectively (Figure 3E).

Finally, ROC analysis in AO patients and EO patients was applied using their respective age-matched healthy subjects for adjustment. As expected, significant AUC values for Valine, Tyrosine, and Leucine were obtained (Tables S3 and S4). Furthermore, decreased Methionine levels in EO patients were confirmed, compared with AO patients and age-matched healthy subjects (Figure S1). However, ROC curves from AC14OH and AC18OH showed no significant *p* values, probably due to that EO and AO samples were not large enough to maintain significant adjusted *p* values (Tables S3 and S4).

## 4. Discussion

Metabolomics profiling is a promising strategy for identifying relevant metabolites/biomarkers in neurodegenerative pathologies [32,33]. Since the current evaluation of SCA7 patients is based on Brief Ataxia Rating Scale (BARS), SARA, and INAS scores [3], time-consuming analyses that require extensive examiner training to avoid bias, identification of sensitive biomarkers will surely improve the clinical management of these patients.

In this study, we show for the first time, a metabolomics profile of circulating free carnitine, ACCs, and AAs in SCA7 patients. These metabolites are involved in mitochondrial function and oxidative stress, two cellular processes that are altered in SCA7 [13,15,16], and other neurodegenerative disorders, including Alzheimer’s Disease (AD), Parkinson Disease (PD), and Huntington Disease (HD) [34,35,36]. Interestingly, significantly lower levels of branched-chain amino acids (BCAAs; Valine and Leucine) and Tyrosine were found in the plasma samples of SCA7 patients. To our knowledge, the association of BCAAs with the pathophysiology of SCA7 has not been observed previously. Thus, we were prompted to analyze whether the assessment of these amino acids could have a diagnostic power in SCA7. A predictive model based on the plasma concentrations of Valine, Leucine, and Tyrosine, as well as the main clinical variables of the disease, was set up. Remarkably, this model had the ability to distinguish between SCA7 patients and healthy control with a sensitivity of 60% and specificity of 86.7%, implying that measurement of the concentration of these amino acids in plasma could serve as a biomarker in SCA7. Interestingly, depleted levels of BCAAs are associated with neurodegeneration [37], an increased risk of dementia [38], and with a faster cognitive decline and significant cerebral atrophy changes in AD [39]. Furthermore, weight loss and sarcopenia have been linked with a deficiency of BCAAs [40].

Concerning the physiological relevance of AAs deficiency in cerebellar ataxias, decreased concentrations of Valine, Leucine, and aromatic AAs (Tryptophan and Tyrosine) were recently reported in the serum of SCA3 patients [41]. Since BCAAs serum levels are largely determined by dietary protein intake [42], reduced plasma levels of these essential AAs in SCA7 patients might indicate a subclinical nutritional deficiency. Consistent with this idea, supplementation of BCAA alleviated cerebellar symptoms in SCA6 patients [43], while Tyrosine administration improved memory and cognitive function in SCA3 patients [41]. Nevertheless, our enrichment analysis of metabolic pathways suggests enhanced catabolism of BCAAs in SCA7; thus, deficiency of BCAAs in patients could not be overcome only by dietary management. The enrichment analysis also revealed defective ammonia recycling in SCA7. Since ammonia easily crosses the blood-brain barrier, blood-derived ammonia leads to neurotoxic levels of ammonia in the brain [44]. Aside from primarily affecting the brain, the toxicity of ammonia has also been demonstrated to affect other organs and tissues, including muscle. Muscle plays a significant role in the ammonia-removing pathway during the amination of glutamate to glutamine. Therefore, muscle mass depletion further reduces the body’s capacity to clear ammonia, which in turn leads to a higher risk of developing hyperammonemia. Interestingly, both HD and SCA7 patients have elevated plasma ammonia levels [44], therefore it is tempting to speculate that dysregulated brain energy metabolism in SCA7 patients could be facilitated, at least in part, through imbalances in ammonia homeostasis.

An additional goal of this study was to identify metabolites with the ability to discriminate between SCA7 phenotypes (EO and AO) because stage-specific biomarkers would allow monitoring the natural history of SCA7, as well as the response of patients under clinical trials. We found a differential content of AC18OH, AC14OH, and Methionine between EO and AO patients; EO patients exhibited significantly lower content of AC18OH and Methionine and higher levels of AC14OH, compared to AO patients. We then established a predictive model with the power to discriminate between EO and AO patients, which considered this metabolite signature and the main SCA7 clinical features. Concerning the physiological relevance of these metabolites, Methionine plays a critical role as an antioxidant in metabolism, immunity, and cell physiology [45,46,47]; indeed, Methionine has been shown to chelate lead and remove it from tissues, which decreases oxidative stress [48]. It is worth to note that a direct link between mutant ataxin-7 aggregation and oxidative stress was observed in a PC12 cell-based model for SCA7. These authors reported that expression and aggregation of mutant ataxin-7 resulted in increased reactive oxigen species (ROS) levels and decreased levels of catalase, a key detoxifying enzyme [13]. Furthermore, patients with SCA7 exhibited elevated levels of oxidative stress markers in circulation, and deregulation of the redox system correlates [15]. Thus, Methionine deficiency could lead to oxidative stress, a pathological feature of SCA7 [13,15].

Furthermore, Methionine has been implicated in lipid metabolism, because Methionine restriction can reduce fat accumulation by caloric restriction, which increases de novo lipogenesis, lipolysis, and fatty acid oxidation [46]. Interestingly, dietary Methionine restriction led to decreased amyloid-beta levels and neuroprotection in APP-PS1 AD mice [49]. On the other hand, AC18OH and AC14OH have been associated with mitochondrial dysfunction and -oxidation [50,51,52]; however, dysregulation in the content of these metabolites appears to be specific of SCA7, because there is no report of such alteration in other polyQ diseases. Therefore, this dysregulation could reflect altered mitochondrial oxidative metabolism [16]. Recent evidence demonstrated impaired mitochondrial function in patients with SCA7 and a murine SCA7 model [16]. Patients with SCA7 were unable to increase ATP production in the visual cortex during the completion of a visual task, which reflects altered mitochondrial oxidative metabolism. Furthermore, SCA7 266Q knock-in mice exhibited mitochondrial fragmentation in cerebellar Purkinje cells and dendrites [16]. Remarkably, reduced NAD+ production in the nucleus and low expression of the *NMNAT1* gene (the main gene involved in NAD+ production) were found in SCA7 neural progenitor cells (NPC), which might result in decreased NAD+ availability in mitochondria; decreased levels of NADH can consequently disturb the electron donor system that drives oxidative phosphorylation at the inner mitochondrial membrane [16]. Finally, perturbation of the tryptophan-kynurenine pathway, which is upstream of the NAD+ precursor de novo synthesis, was found in the plasma samples of SCA7 patients [16]. Disturbance of the synthesis of NAD+ could impact multiple pathways, such as catabolism of fatty acids (FAs). Therefore, enrichment analysis points to fatty acids and carnitine metabolism as ACCs-related metabolic pathways involved in SCA7. These processes take place in mitochondria, where the role of ACCs is to transport long-chain fatty acids into mitochondria for -oxidation [52]; in fact, fatty acids have been implicated in diverse neurodegenerative diseases [50,51,53]. Furthermore, ACCs have been shown to possess diverse neuroprotective effects, including improvement of mitochondrial function, modulation of gene expression, enhancement of cholinergic neurotransmission, antioxidant activity, and membrane stabilization [18,23,45,54,55]. Thus, metabolic changes in these metabolites could be an indicator of these mechanistic alterations. It should be noted that the analysis of our study population is highly valuable due to the rare worldwide incidence of SCA7 (<1/100,000) [8,18]. Future longitudinal studies on larger samples of patients, as well as comparative analyses of SCA7 with other SCAs and polyQ diseases, are required to confirm the existence of disease- and even disease-stage-specific metabolites in SCA7.

## 5. Conclusions

We carried out a metabolomics profiling of ACCs, free carnitine, and AAs in the plasma samples of SCA7 patients. We identified promising metabolites that could serve as auxiliary biomarkers for the diagnostic and prognosis of the disease because they are sampled in a relatively non-invasive manner and are readily detected by easy biochemical assays that could be implemented in clinical laboratories.

## Figures and Tables

**Table 1:** Demographic and Clinical Features of Studied subjects.

		Control	Patients with SCA7	Patients with SCA7
	Patients with SCA7	Healthy Subjects	Adult Onset (AO)	Early Onset (EO)
N	20	20	10	10
Female/Male	10/10	10/10	4/6	6/4
Age	41.95 ± 12.62	43.95 ± 12.8	53.2 ± 6.2	30.7 ± 5.8
Visual: age at onset	29.9 ± 12.50	NA	41.2 ± 7.5	18.6 ± 2.6
Motor: age at onset	31.35 ± 10.75	NA	40.8 ± 6.9	21.9 ± 3.2
First symptom age	29.05 ± 12.11	NA	40 ± 7.4	18.1 ± 2.1
CAG Repeats	46.15 ± 4.25	10.4 ± 0.8	42.5 ± 1.9	49.8 ± 2.5
SARA	18.15 ± 8.03	NA	17.2 ± 8.6	19.1 ± 8.2
INAS	4.6 ± 2.31	NA	4.6 ± 2.3	4.6 ± 2.5

NA. Not applicable. SCA 7: Spinocerebellar Ataxia type 7. SARA: Scale for the Assessment and Rating of Ataxia; INAS: Inventory of Non-Ataxia Symptoms.

## Supplementary Materials

The following are available online at https://www.mdpi.com/2218-273X/10/3/390/s1, Table S1: Differential metabolites profile between patients with SCA7 and control subjects, Table S2: Differential profile of circulating metabolites between EO and AO patients with SCA7, Table S3: Differential metabolites profile between patients with EO SCA7 and age- matched control subjects, Table S4: Differential metabolites profile between patients with AO SCA7 and age- matched control subjects, Figure S1: Methionine levels in EO and AO patients.
