# Supplementary material for: Altered Plasma Acylcarnitines and Amino Acids Profile in Spinocerebellar Ataxia Type 7
Source: Biomolecules. 2020 Mar 3;10(3):390. doi: 10.3390/biom10030390 (PMC7175318; doi:10.3390/biom10030390)
Supplement: Supplementary file 1 [file biomolecules-10-00390-s001.zip › Supplementary files/Supp Tables.docx]

**Supplementary Table 1.** Differentially metabolites profile between patients with SCA7 and control subjects

| Metabolite | AUC (basal) | T-tests | Log2 FC |
| --- | --- | --- | --- |
| VAL | 0.9725 | 5.232E-10 | 0.38982 |
| LEU | 0.945 | 1.6248E-8 | 0.33125 |
| TYR | 0.93 | 5.51E-7 | 0.38944 |
| PHE | 0.8875 | 1.7035E-5 | 0.19601 |
| C0 | 0.855 | 4.5648E-5 | 0.26573 |
| C5 | 0.8275 | 7.2032E-5 | 0.1917 |
| C18/1OH | 0.82 | 5.9928E-4 | 0.042786 |
| ALA | 0.8075 | 3.2889E-4 | 0.14454 |
| MET | 0.8 | 5.4933E-4 | 0.070948 |
| SA | 0.795 | 0.0010129 | 0.14413 |
| C5/1 | 0.7825 | 0.0020077 | 0.10697 |
| C3 | 0.7475 | 0.0061367 | 0.22655 |
| C16OH | 0.7425 | 0.0067355 | 0.068107 |
| C16 | 0.7325 | 0.01515 | 0.088746 |
| C6DC | 0.725 | 0.0064442 | 0.19288 |
| GLY | 0.715 | 0.04328 | -0.38866 |
| C4 | 0.715 | 0.028349 | 0.09851 |
| ARG | 0.7025 | 0.056412 | 0.0068527 |
| C16/1OH | 0.69 | 0.078648 | 0.013803 |
| C10/2 | 0.69 | 0.020256 | 0.059066 |
| PRO | 0.685 | 0.034805 | 0.025735 |
| C18 | 0.675 | 0.084758 | 0.042486 |
| C14 | 0.67 | 0.08892 | 0.027605 |
| C14OH | 0.6675 | 0.093209 | -0.013803 |
| ORN | 0.655 | 0.034049 | 0.061685 |
| AGE | 0.655 | 0.042508 | 0.081869 |
| C2 | 0.64 | 0.10377 | 0.19935 |
| C18OH | 0.6275 | 0.138 | 0.007131 |
| C8/1 | 0.6125 | 0.25337 | -0.0081834 |
| C18/1 | 0.605 | 0.33323 | -0.019016 |
| C16/1 | 0.5875 | 0.41836 | -0.008743 |
| C6 | 0.585 | 0.2715 | -0.017188 |
| C18/2 | 0.58 | 0.43953 | -0.014881 |
| C12 | 0.5675 | 0.47412 | -0.041433 |
| C12/1 | 0.5625 | 0.38132 | -0.074943 |
| C14/2 | 0.5575 | 0.47819 | -0.12753 |
| C8 | 0.5475 | 0.50662 | -0.26691 |
| C10/1 | 0.54 | 0.48922 | -0.096676 |
| C10 | 0.5375 | 0.74696 | -0.20478 |
| C14/1 | 0.5075 | 0.82429 | -0.15785 |

**Supplementary Table 2.** Differential profile of circulating metabolites between EO and AO patients with SCA7.

| VARIABLE | AUC (BASAL) | T-tests | Log2 FC |
| --- | --- | --- | --- |
| AGE OF VISUAL SYMPTOMS | 1.0 | 1.0687E-6 | 1.1395 |
| AGE AT ONSET | 0.99 | 7.6492E-7 | 1.1327 |
| AGE OF MOTOR SYMPTOMS | 0.96 | 2.8181E-4 | 0.89966 |
| DISEASE TIME SPAN | 0.95 | 5.4245E-4 | 0.82176 |
| C14OH | 0.76 | 0.038645 | 0.027605 |
| MET | 0.75 | 0.096112 | 0.1268 |
| C18OH | 0.72 | 0.23906 | 0.014262 |
| SARA | 0.72 | 0.11421 | -0.23689 |
| C16/1OH | 0.71 | 0.4933 | 0.055211 |
| C12/1 | 0.71 | 0.11114 | 0.060072 |
| ALA | 0.7 | 0.10213 | 0.12158 |
| C14 | 0.68 | 0.13451 | 0.19143 |
| C10/1 | 0.68 | 0.17271 | 0.085174 |
| C4 | 0.67 | 0.1739 | 0.47582 |
| C16OH | 0.67 | 0.51453 | 0.055211 |

**Supplementary Table 3.** Differentially metabolites profile between patients with EO SCA7 and age- matched control subjects

| METABOLITE | AUC (BASAL) | T-tests | Log2 FC |
| --- | --- | --- | --- |
| VAL | 0.84 | 0.0079321 | 0.4177 |
| LEU | 0.8 | 0.012801 | 0.37925 |
| C12 1 | 0.74 | 0.045213 | -0.11271 |
| GLY | 0.72 | 0.078923 | -0.1305 |
| C14 1 | 0.71 | 0.08207 | -0.19613 |
| TYR | 0.66 | 0.09933 | 0.3348 |
| C0 | 0.71 | 0.1426 | 0.38723 |
| C10 1 | 0.69 | 0.15589 | -0.12982 |
| C14 2 | 0.68 | 0.15782 | -0.17587 |
| C12 | 0.63 | 0.19731 | -0.11955 |
| C10 | 0.64 | 0.19882 | -0.2918 |
| C18 1OH | 0.62 | 0.20558 | 0.057048 |
| C5 1 | 0.64 | 0.22283 | 0.15839 |
| CIT | 0.65 | 0.30109 | -0.014309 |
| C8 | 0.62 | 0.30725 | -0.25193 |
| C16 1 | 0.67 | 0.3093 | -0.053399 |
| C5 | 0.6 | 0.33981 | 0.17663 |
| C8 1 | 0.63 | 0.35803 | -0.10336 |
| C16 | 0.6 | 0.36188 | 0.14611 |
| C4 | 0.63 | 0.37017 | 0.23118 |
| C3 | 0.6 | 0.39601 | 0.26305 |
| C18 2 | 0.66 | 0.40726 | -0.027605 |
| C6DC | 0.63 | 0.42199 | -0.011268 |
| ALA | 0.62 | 0.43154 | 0.22452 |
| SA | 0.59 | 0.44134 | 0.2451 |
| C18OH | 0.72 | 0.44686 | 0 |
| PRO | 0.57 | 0.45488 | 0.022821 |
| C2 | 0.56 | 0.46892 | 0.41045 |
| ORN | 0.63 | 0.48191 | 0.014826 |
| C10 2 | 0.5 | 0.68076 | 0.11813 |
| ARG | 0.58 | 0.71277 | 0.16839 |
| C18 1 | 0.61 | 0.75632 | 0.0018114 |
| MET | 0.58 | 0.76833 | 0.095244 |
| C6 | 0.56 | 0.79675 | 0.030013 |
| C16OH | 0.6 | 0.8446 | 0.027605 |
| C14OH | 0.57 | 0.87413 | 0.027605 |
| PHE | 0.54 | 0.88265 | 0.13993 |

**Supplementary Table 4.** Differentially metabolites profile between patients with AO SCA7 and age- matched control subjects

| METABOLITE | AUC (BASAL) | T-tests | Log2 FC |
| --- | --- | --- | --- |
| GLY | 0.97 | 1.19E-05 | -0.64683 |
| TYR | 0.94 | 5.81E-04 | 0.44408 |
| VAL | 0.92 | 6.02E-04 | 0.36194 |
| LEU | 0.82 | 0.011011 | 0.28326 |
| C6DC | 0.82 | 0.010822 | 0.39703 |
| CIT | 0.8 | 0.019914 | -0.43523 |
| C5 | 0.78 | 0.020122 | 0.20676 |
| PHE | 0.77 | 0.032719 | 0.25208 |
| ALA | 0.71 | 0.37891 | 0.064551 |
| C0 | 0.7 | 0.1167 | 0.14423 |
| C14OH | 0.69 | 0.54761 | -0.027605 |
| C8 1 | 0.66 | 0.41451 | 0.086989 |
| C16OH | 0.65 | 0.12231 | 0.10861 |
| C3 | 0.63 | 0.28663 | 0.19004 |
| C16 1 | 0.62 | 0.50747 | 0.035913 |
| SA | 0.61 | 0.54623 | 0.043152 |
| C8 | 0.61 | 0.20985 | -0.2819 |
| C14 2 | 0.61 | 0.55081 | -0.079193 |
| MET | 0.6 | 0.24413 | 0.046652 |
| ORN | 0.6 | 0.41145 | 0.10854 |
| C5 1 | 0.58 | 0.29958 | 0.055555 |
| C16 | 0.58 | 0.63961 | 0.031382 |
| C18 | 0.58 | 0.48834 | 0.031573 |
| PRO | 0.57 | 0.65782 | 0.028649 |
| C14 1 | 0.56 | 0.59265 | -0.11958 |
| ARG | 0.55 | 0.46576 | -0.15468 |
| C6 | 0.55 | 0.77816 | -0.064389 |
| C10 | 0.55 | 0.70077 | -0.11775 |
| C18 1 | 0.55 | 0.96939 | -0.039843 |
| C12 | 0.54 | 0.6846 | 0.036687 |
| C16 1OH | 0.53 | 0.58509 | 0.027605 |
| C14 | 0.53 | 0.58539 | 0.027605 |
| C4 | 0.52 | 0.9672 | -0.034164 |
| C10 1 | 0.52 | 0.78876 | -0.06353 |
| C10 2 | 0.52 | 0.80946 | 0 |
| C18 1OH | 0.52 | 0.18918 | 0.028524 |
| C18OH | 0.51 | 0.63301 | 0.014262 |
