# Supplementary figures and images for: Altered Plasma Acylcarnitines and Amino Acids Profile in Spinocerebellar Ataxia Type 7
Source: Biomolecules. 2020 Mar 3;10(3):390. doi: 10.3390/biom10030390 (PMC7175318; doi:10.3390/biom10030390)

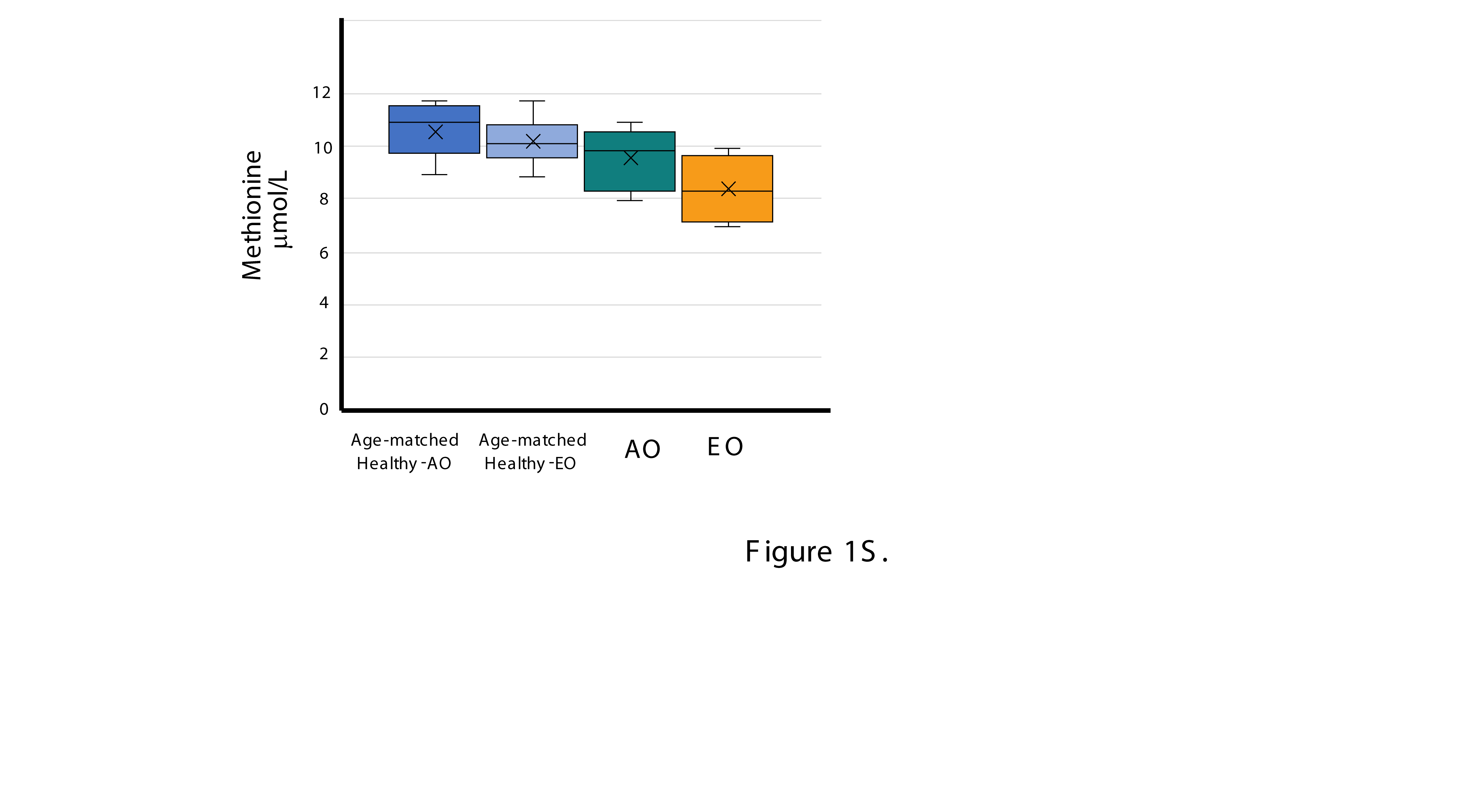

Supplement: Supplementary file 1 [file biomolecules-10-00390-s001.zip › Supplementary files/Figure 1 Suppl.tif]
